# Surgical approaches to colonic and rectal anastomosis: systematic review and meta-analysis

**DOI:** 10.1007/s00384-023-04328-6

**Published:** 2023-02-23

**Authors:** Ana Oliveira, Susana Faria, Nuno Gonçalves, Albino Martins, Pedro Leão

**Affiliations:** 1 Life and Health Sciences Research Institute (ICVS), School of Medicine, University of Minho, Braga, 4710-057 Portugal; 2 ICVS/3B’s-PT Government Associate Laboratory, Braga/Guimarães, Portugal; 3 3B’s Research Group, I3Bs – Research Institute on Biomaterials, Biodegradables & Biomimetics; Headquarters of the European Institute of Excellence on Tissue Engineering & Regenerative Medicine, University of Minho, AvePark-Parque de Ciência e Tecnologia, Zona Industrial da Gandra, Barco, Guimarães, 4805-017 Portugal; 4 Centre of Mathematics (CMAT), Department of Mathematics, University of Minho, Guimarães, 4800-058 Portugal

**Keywords:** Anastomosis, Colon, Rectum, Handsewn, Stapled, Compression

## Abstract

**Purpose:**

Postoperative complications after a colonic and rectal surgery are of significant concern to the surgical community. Although there are different techniques to perform anastomosis (i.e., handsewn, stapled, or compression), there is still no consensus on which technique provides the least number of postoperative problems. The objective of this study is to compare the different anastomotic techniques regarding the occurrence or duration of postoperative outcomes such as anastomotic dehiscence, mortality, reoperation, bleeding and stricture (as primary outcomes), and wound infection, intra-abdominal abscess, duration of surgery, and hospital stay (as secondary outcomes).

**Methods:**

Clinical trials published between January 1, 2010, and December 31, 2021, reporting anastomotic complications with any of the anastomotic technique were identified using the MEDLINE database. Only articles that clearly defined the anastomotic technique used, and report at least two of the outcomes defined were included.

**Results:**

This meta-analysis included 16 studies whose differences were related to the need of reoperation (*p* < 0.01) and the duration of surgery (*p* = 0.02), while for the anastomotic dehiscence, mortality, bleeding, stricture, wound infection, intra-abdominal abscess, and hospital stay, no significant differences were found. Compression anastomosis reported the lowest reoperation rate (3.64%) and the handsewn anastomosis the highest (9.49%). Despite this, more time to perform the surgery was required in compression anastomosis (183.47 min), with the handsewn being the fastest technique (139.92 min).

**Conclusions:**

The evidence found was not sufficient to demonstrate which technique is most suitable to perform colonic and rectal anastomosis, since the postoperative complications were similar between the handsewn, stapled, or compression techniques.

## Introduction

Anastomotic dehiscence after colonic and rectal resection is a dreaded complication with increased morbidity and significant mortality rate, ranging from 6 to 22% depending on the anastomotic site [1]. The consequences of an ineffective surgery can be so diverse and devastating that, in addition to the fearsome anastomotic leakage, this situation can also cause the appearance of bleeding, strictures, and intra-abdominal and wound infections [2–5]. Late diagnosis can even lead to cases of generalized peritonitis progressing to sepsis, compromising the patient’s life. It may require reoperation and, consequently, an increase in hospital stay with inevitable extra economic costs [2, 6].

Several aspects have been identified as possible predictors of anastomotic complications, being divided in patient-related risk factors [7–10] and surgical procedure’s characteristics [11, 12]. Focusing on the surgery itself, different techniques can be used to perform colonic and rectal anastomosis, namely handsewn, stapled, or compression. The handsewn and stapled techniques are the most commonly used, although associated with the idea that the introduction of foreign materials can injure the intestinal tissue and trigger an inflammatory response [2, 13, 14]. Despite handsewn anastomosis be a traditional technique, the stapled anastomosis has become very attractive due to its ease implementation. By its side, compression anastomosis involves the use of devices, such as clips and rings, to perform an end-to-end sutureless anastomosis. The intestinal segments are compressed by these devices that place the ends in apposition. Afterwards, the devices are expelled spontaneously by the body. Although compression anastomosis is considered safe, it has not yet achieved considerable popularity among the surgical community [15–19]. Despite all those scientific evidences, there is no consensus concerning the most advantageous technique to perform a colonic and rectal anastomosis. Despite different technical characteristics and handling skills, there is no specific guidance regarding the technique that should be used, and these decisions have been based on the surgeon’s experience and preference [5, 14, 18, 20]. Therefore, studies comparing the three anastomotic techniques independently are needed to guide the medical community towards the most suitable technique. In addition, these analyses should not be restricted to studies that compare more than one technique, otherwise a large part of the sample will not be included.

In this systematic review and meta-analysis, we intended to understand which technique is the most successful to perform a colonic and rectal anastomosis, providing fewer postoperative complications.

## Methods

### Search strategy

A literature search was conducted using the MEDLINE database for studies published between January 1, 2010, and December 31, 2021, using the following combinations of keywords:

1. colon AND anastomosis AND dehiscence
2. colon AND anastomosis AND suture
3. colon AND anastomosis AND infection
4. colon AND anastomosis AND inflammation

The search was restricted to English language publications describing clinical investigations in humans, more specifically, clinical trials. The studies included comply with the Preferred Reporting Items for Systematic Reviews and Meta-analysis (PRISMA) methodology [21].

### Data collection and analysis

Three authors selected the studies independently. The data were extracted and verified independently by each one. The final data were combined and analyzed.

### Types of outcome measures

Anastomotic dehiscence, mortality, reoperation, bleeding, and stricture represented the primary outcomes, while wound infection, intra-abdominal abscess, duration of surgery, and hospital stay were defined as secondary outcomes.

#### Primary outcomes

- Anastomotic dehiscence: dehiscences or leaks diagnosed clinically through the discharge of feces at the anastomosis site, identified radiologically, through the presence of leakage with the postoperative control enema in a patient who had no evidence of a clinical anastomotic leakage, or by operative confirmation.
- Mortality: postoperative deaths due to anastomotic complications.
- Reoperation: surgical reintervention due to an anastomotic complication that cannot be treated conservatively.
- Bleeding: postoperative bleeding or hemorrhage that occurs from the anastomotic site or in the abdominal cavity.
- Stricture: narrowing in the intestinal lumen as a result of anastomotic healing.

#### Secondary outcomes

- Wound infection: the presence of infection in the abdominal wound.
- Intra-abdominal abscess: the accumulation of fluids in the abdominal cavity.
- Duration of surgery: time to perform the entire surgical procedure, including anastomosis.
- Hospital stay: time from surgery to hospital discharge.

### Inclusion criteria

Only articles that clearly defined the type of anastomosis used (handsewn, stapled, or compression), and report at least two of the outcomes defined above.

### Exclusion criteria

Exclusion criteria were (1) procedures without anastomosis of the colon or rectum; (2) emergency surgery or no clarification on its origin; (3) investigation of new diagnostic methods; (4) approaches that deviate from clinical practice in the handsewn and stapled techniques, by implementing new methods, therapies or devices; and (5) no results for at least two study outcomes.

### Data extraction

Some data was extracted from the studies, such as first author and year of publication, trial registration, study design, time of perspective, randomization, number of patients included, objective, study groups, age and sex of the patients, type of anastomosis, anastomotic site, procedure, surgical approach, characteristics of anastomosis, diagnostic methods, other relevant procedures, bowel preparation and prophylaxis, endpoints of the study, and main results. The total number of patients who respond to the outcomes defined above were also extracted, as well as the duration of surgery and length of hospital stay.

### Statistical analysis

The meta-analysis was conducted using the “meta” package (version 4.18.0, 2021) in R (The R Foundation for Statistical Computing; version 4.0.4, 2021).

For the duration of surgery and hospital stay outcomes, results are presented as means with an associated 95% confidence interval (CI). Study data presented as median and ranges were converted to mean and standard deviation (SD) using the method developed by Wan et al. [22]. For the other outcomes, the results are presented as proportions with associated 95% CI. The Mantel–Haenszel statistical method was applied for dichotomous outcomes. Continuous outcomes were analyzed using the mean difference (MD) with an associated 95% CI and pooled using an inverse variance model.

The subgroup analysis was performed based on the type of anastomotic technique (handsewn, stapled, or compression). Studies in which the outcome of interest was not observed in either group were excluded from the meta-analysis of that income. *p* < 0.05 was considered to indicate statistical significance.

Sensitivity analysis was performed by excluding one study from the data set to investigate its influence on the overall results, and explore sources of significant heterogeneity. Considering the heterogeneity of the clinical studies, which refers to diversity relevant to complicated clinical situations, we used the random-effects model based on the Sidik-Jonkman method [23]. An inverse-variance random-effects model was used for all analyses. Heterogeneity between studies was assessed using the *I*^2^ statistics (heterogeneities < 25%, 25–50%, and > 50% were considered as low, moderate, and high, respectively [24]) and Chi-square test (${\chi }^{2}$), with *p* < 0.05 considered statistically significant.

Tests for funnel plot asymmetry were used in each outcome when there were at least ten studies included in the meta-analysis [25]. Egger’s test was used to assess potential publication bias via funnel plost asymmetry.

## Results

**Fig. 1:**
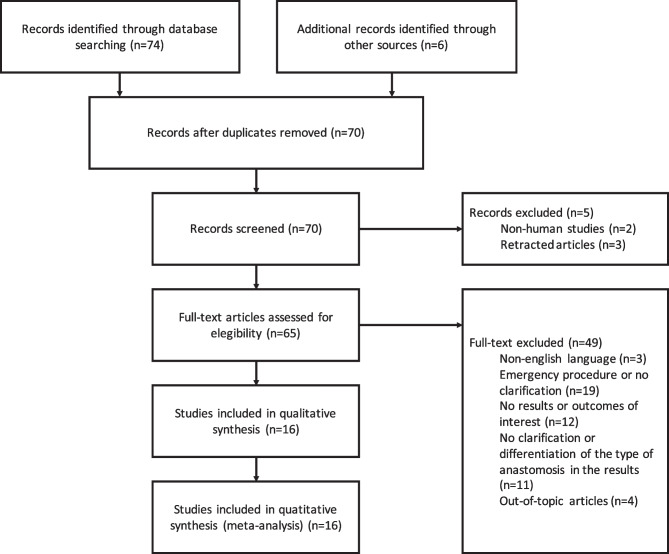
PRISMA flowchart: selection of relevant studies

The literature search identified a total of 74 studies. Of these studies, 14 corresponded to the first combination of keywords, 14 to the second, 40 to the third, and 6 to the fourth. Additionally, six articles were included through the analysis of reference lists. However, ten duplicate studies were removed, leaving a total of 70 studies for screening according to Fig. 1. In this analysis, 16 studies were included, comprising a total of 7259 patients. Of these, two studies had a handsewn anastomosis, seven stapled, three by compression, and four included both handsewn and stapled. Overall, 3513 patients underwent handsewn anastomosis, 3417 stapled, and 329 compression. The reinforcement groups of the studies included in the stapled technique were not included, and the analysis was restricted to 3079 patients. The characteristics of studies and patient demographics are shown in Table 1.

**Table 1:** Characteristics and patient demographic data of the included studies

**Source**	**Trial registration**	**Study design**	**Time of perspective**	**Randomization**	**Number of cases**	**Objective**	**Groups**	**Age (years), mean ± SD**	**Sex,** ***n***		**Type of anastomosis**
									**Male**	**Female**	
Ferrer-Márquez et al. (2021) [26]	NTC03990714	Multicenter	Prospective	Yes	160	Evaluation of short-term outcomes of performing intracorporeal anastomosis (IA) versus extracorporeal anastomosis (EA) in laparoscopic right hemicolectomy for right colon neoplasm	IA (*n* = 82); EA (*n* = 78)	IA: 70.51 ± 9.88; EA: 68.65 ± 12.51	IA: 43; EA: 39	IA: 39; EA: 39	Stapled
Milone et al. (2021) [27]	NCT03422588	Single institution	Prospective	Yes	59	Evaluation of the surgical stress response and the metabolic response in patients who underwent right colonic resection for colon cancer	IA (*n* = 30); EA (*n* = 29)	IA: 65.26 (4.42); EA: 66.30 (4.19)	IA: 13; EA: 13	IA: 17; EA: 16	Stapled
Mai-Phan et al. (2019) [28]	-	Non-blind, single center	Prospective	Yes	122	Evaluation of the effect of mechanical bowel preparation (MBP) on elective laparoscopic colectomy	MBP (*n* = 62); No-MBP (*n* = 60)	MBP: 57.0 ± 14.8; No-MBP: 58.2 ± 14.3	MBP: 27; No-MBP: 33	MBP: 35; No-MBP: 27	Handsewn
Jurowich et al. (2019) [29]	-	-	Retrospective	No	4062	Assessment whether the anastomotic technique (handsewn or stapled) after open right hemicolectomy for right-sided colonic cancer influences postoperative complications	Handsewn (*n* = 2742); Stapled (*n* = 1320)	Handsewn: 72.9 ± 10.9; Stapled: 73.9 ± 10.6	Handsewn: 1293; Stapled: 622	Handsewn: 1449; Stapled: 698	Handsewn and stapled
Bakker et al. (2017) [30]	NTR3080	Non-blind, multicenter	Prospective	Yes	402	Evaluation of the efficacy of the C-seal in reducing anastomotic leakage in stapled colorectal anastomosis	C-seal (*n* = 202); No C-seal, Control (*n* = 200)	C-seal: 66; Control: 64	C-seal: 121; Control: 120	C-seal: 81; Control: 80	Stapled
Herrle et al. (2016) [3]	NCT00996554	Double-blind, multicenter	Prospective	Yes	252	Evaluation of the complication rates after hand-sutured continuous single-layer anastomosis (SLA) with continuous double-layer colo-colonic and ileocolonic anastomosis (DLA) in elective colorectal surgery	SLA (*n* = 129); DLA (*n* = 123)	SLA: 67.7 ± 11.1; DLA: 64.6 ± 13.4	SLA: 72; DLA: 69	SLA: 57; DLA: 54	Handsewn
Frasson et al. (2016) [31]	-	Observational, multicenter	Prospective	No	1102	Determination of pre-/intraoperative risk factors for anastomotic leakage after elective right colon resection for cancer	Handsewn (*n* = 324); Stapled (*n* = 778)	74 (66–80)	622	480	Handsewn and stapled
Matsuda et al. (2015) [32]	MIN00000848	Single-blind, single center	Prospective	Yes	40	Evaluation of short-term outcomes of isoperistaltic stapled side-to-side anastomosis (SSSA) comparing them with antiperistaltic SSSA during colon cancer surgery	Isoperistaltic SSSA (*n* = 20); Antiperistaltic SSSA (*n* = 20)	Antiperistaltic SSSA: 68 ± 10; Isoperistaltic SSSA: 66 ± 12	Antiperistaltic SSSA: 11; Isoperistaltic SSSA: 11	Antiperistaltic SSSA: 9; Isoperistaltic SSSA: 9	Stapled
D’Hoore et al. (2015) [4]	NCT01091155	Non-blind, multicenter	Prospective	No	266	Evaluation of ColonRing™ performance in (low) colorectal anastomosis	-	62	138	128	Compression
Placer et al. (2014) [33]	-	Single-blind, single center	Prospective	Yes	281	Evaluation of the effectiveness of bioabsorbable staple line reinforcement (BSLR) in reducing colorectal anastomotic complications	BSLR (*n* = 136); Control (*n* = 145)	BSLR: 67; Control: 66	BSLR: 88; Control: 90	BSLR: 48; Control: 55	Stapled
Leung et al. (2013) [34]	-	Double-blind	Prospective	Yes	70	Comparison of the short-term outcomes of patients who underwent hybrid NOTES colectomy (HNC) with those who underwent conventional laparoscopic colectomy (CL)	HNC (*n* = 35); CL (*n* = 35)	HNC: 62; CL: 72	HNC: 13; CL: 12	HNC: 22; CL: 23	Stapled
Khromov et al. (2013) [35]	-	Non-blind, multicenter	Prospective	No	40	Evaluation of the short-term clinical outcome and safety profile of the NiTi Biodynamix ColonRing™ compression anastomosis in elective colorectal resection	-	65.8	18	22	Compression
Ruiz-Tovar et al. 2012 [36]	NCT01458353	Blinded for results analysis, single center	Prospective	Yes	84	Prospective evaluation of the peritoneal contamination after the performance of stapled or handsewn anastomosis and if these data correlate with a surgical-site infection	Stapled (*n* = 42); Handsewn (*n* = 42)	Stapled: 68.5 ± 10.2; Handsewn: 69.9 ± 11.5	Stapled: 26; Handsewn: 25	Stapled: 16; Handsewn: 17	Handsewn and stapled
Zurbuchen et al. (2013) [37]	ISRCTN-45665492	Single-blind, multicenter	Prospective	Yes	67	Investigation whether stapled side-to-side anastomosis, compared to the handsewn end-to-end anastomosis, results in a decreased recurrence of Crohn’s disease following ileocolic resection	Side-to-side (*n* = 36); end-to-end (*n* = 31)	Side-to-side: 39.5 ± 12.55; end-to-end: 39.1 ± 12.58	Side-to-side: 17; end-to-end: 19	Side-to-side: 19; end-to-end: 12	Handsewn and stapled
Bertani et al. (2011) [38]	-	Single center	Prospective	Yes	229	Evaluation of the impact of preoperative MBP for colon and rectal cancer surgery in comparison with a single glycerine enema	Preoperative MBP plus a glycerine 5% enema, MBP (*n* = 114); Single glycerine 5% enema, no-MBP (*n* = 115)	MBP: 63; no-MBP: 64	MBP: 65; no-MBP: 60	MBP: 49; no-MBP: 55	Stapled
Buchberg et al. (2011) [18]	-	Pilot study, single center	Prospective	No	23	Evaluation of the outcomes of the NiTi CAR™ 27 compression anastomosis device in patients undergoing a left-sided colectomy	-	60	9	14	Compression

Studies reporting emergency surgeries were not considered due to the higher rates of anastomotic complications and because they did not provide relevant information regarding mechanical bowel preparation and additional prophylaxis. Studies investigating new diagnostic techniques were not included due to the impossibility of knowing which technique is most effective in detecting anastomotic complications and which results should be considered. As shown, only four studies compare different types of anastomosis and none makes a global comparison of all techniques. For this reason, this analysis covered not only comparative studies but also studies evaluating isolated anastomotic techniques.

### Techniques of colonic and rectal anastomosis

Each study was analyzed for the outcomes reported, with the overall results shown in Table 2. Since the studies reported different outcomes, each outcome was calculated for each type of anastomosis, according to the number of patients in the study who respond to it.

**Table 2:** Main characteristics and results of included studies

**Source**	**Site**	**Procedure**	**Surgical approach**	**Characteristics of anastomosis**	**Diagnostic methods**	**Other procedures**	**Bowel preparation and prophylaxis**	**Study endpoints**	**Main results**
Ferrer-Márquez et al. (2021) [26]	Colon	Right hemicolectomy for right colon neoplasm	Laparoscopy	Stapled (side-to-side)	-	-	No bowel preparation; intravenous antibiotics preoperatively	Paralytic ileus, surgical-site infection, hospital stay, anastomotic leakage, repeat interventions, postoperative pain	Median of hospital stay: 7 days (both groups). Paralytic ileus: 20.63%. Surgical-site infection: 10% (3.65% IA, 16.67% EA). Anastomotic leakage: 6.25%
Milone et al. (2021) [27]	Colon	Elective	Laparoscopy	Stapled (extracorporeal side-to-side isoperistaltic anastomosis or side-to-side isoperistaltic anastomosis)	-	-	Not mentioned	Levels of IL-6, C-reactive protein (CRP), IL-1β, IL-10, IL-13, tumor necrosis factor α (TNFα), cortisol and insulin	Proinfammatory mediator IL-6, CRP, TNF and IL-1β levels were significantly reduced in IA compared to EA. An improved profile of the anti-inflammatory cytokines IL-10 and IL-13 was observed in the IA group. Cortisol was increased in EA, while insulin was reduced in the EA group
Mai-Phan et al. (2019) [28]	Colon	Elective colon surgery	Laparoscopy	Handsewn (1 and 2 layers)	CT scan, laparotomy, ultrasonography, clinical examination, lab tests, imaging tests	-	MBP (sodium phosphate or polyethylene glycol); intravenous antibiotic prophylaxis	Anastomotic leakage, surgical-site infection, extra-abdominal complications, hospital stay, death	Abdominal complications (anastomotic leak and surgical-site infection): 16.2% MBP, 18.3% no-MBP. Anastomotic leakage: 6.5% MBP, 3.3% no-MBP. Hospital stay: 9.0 ± 2.9 MBP, 8.4 ± 1.9 no-MBP
Jurowich et al. (2019) [29]	Colon	Elective and open right hemicolectomy for right-sided colonic cancer	Laparotomy	Handsewn; Stapled (any type of stapler device)	-	-	Not mentioned	Anastomotic leakage, postoperative ileus, reoperation, surgical-site infection, hospital stay, death	Anastomotic leakage: 3.9% handsewn, 3.0% stapled. Postoperative ileus: 4.0% handsewn, 3.6% stapled. Reoperation: 9.6% handsewn, 10.5% stapled. Superficial site infection: 10.2% handsewn, 9.5% stapled. Postoperative bleeding: 1.8% handsewn, 1.6% stapled. Duration of surgery: 134.1 ± 49.0 handsewn, 120.5 ± 46.5 stapled. Hospital stay: 13.4 ± 9.2 handsewn, 13.6 ± 9.5 handsewn. Mortality: 2.9% handsewn, 3.6% stapled
Bakker et al. (2017) [30]	Colorectal	Elective surgery with a stapled colorectal anastomosis	Laparotomy; laparoscopy; conversion	Stapled (circular; C-seal: biodegradable polyurethane; end-to-end, end-to-side, side-to-end, side-to-side)	Air leak test	-	Mechanical oral bowel preparation	Anastomotic leakage	Overall anastomotic leakage: 7.7%. Anastomotic leakage: 10.4% C-seal, 5.0% control
Herrle et al. (2016) [3]	Colorectal	Elective colorectal surgery	Laparotomy; laparoscopy; conversion	Handsewn (2 layers; continuous; 4–0, 5–0 polydioxanone, ETHICON; 1 layer, 4–0 PDS, ETHICON; end-to-end, end-to-side)	Endoscopy, CT scan, reoperation	-	Not mentioned	Clinical anastomotic leakage, duration of anastomotic suture, morbidity, stool patterns	Clinical anastomotic leakage: 3.1% SLA, 4.9% DLA (not significant - NS). Suture duration: 18 min SLA, 24 min DLA. Subjective well-being and stool patterns: NS
Frasson et al. (2016) [31]	Colon	Elective right colon resection for cancer	Laparotomy; laparoscopy; conversion	End-to-end, end-to-side, side-to-end, side-to-side	-	Suture reinforcement with allogenic or synthetic material was performed on 0.3% of patients	Not mentioned	Preoperative risk factors for anastomotic leakage, wound infection, morbidity, mortality	Anastomotic leakage: 8.4%. Radiological/surgical intervention: 6.5%. Morbidity: 29.0%. Mortality: 2.6%. Wound infection: 13.4%
Matsuda et al. (2015) [32]	Colon	Elective curative resection after colon cancer	Laparotomy; laparoscopy	Stapled (linear, Echelon Endopath; side-to-side)	-	Additional hand sutures for reinforcement (1 layer, 4–0 PDS II, ETHICON)	MBP (polyethylene glycol); intravenous antibiotic prophylaxis (flomoxef sodium)	Anastomotic leakage, hemorrhage, stenosis, wound infection, prolonged ileus, intra-abdominal abscess, first defecation after surgery, reoperation, hospital stay	Anastomotic leakage: 2 isoperistaltic SSSA, 0 antiperistaltic SSSA (NS, *p* = 0.487). Anastomotic stenosis: 1 antiperistaltic SSSA, 0 isoperistaltic SSSA. No anastomotic hemorrhage in either group. Hospital stay: NS
D’Hoore et al. (2015) [4]	Colorectal	Open or laparoscopic left‐sided colorectal resection with the creation of an anastomosis	Laparotomy; laparoscopy; conversion	Compression (ColonRing™; end‐to‐end, colonic pouch reconstruction)	Air leak test, contrast insufflation test, endoscopy (rigid or flexible sigmoidoscopy), radiography	Additional reinforcement or reconstruction and/or fecal deviation when leak test was positive	MBP; antibiotic prophylaxis; thrombosis prophylaxis	Anastomotic leakage	Overall anastomotic leakage: 5.3% (3.1% for low anastomosis). Septic anastomotic complications: 8.3% (8.2% of low anastomosis)
Placer et al. (2014) [33]	Colorectal	Left colon resection for a benign or malignant condition and elective colorectal anastomosis	Laparotomy; laparoscopy	Stapled (circular, proximate, BSLR device-Gore Seamguard: synthetic bioabsorbable polyglycolic acid/trimethylene carbonate copolymer fiber; 2 layers; side-to-end)	Air leak test, imaging tests, visual control, colon and tissue doughnut integrity, proctosigmoidoscopy	-	MBP; antibiotic prophylaxis; antithromboembolic prophylaxis; anti-inflammatory prophylaxis (dexamethasone, carbohydrate-rich drink)	Pooled incidences of anastomotic complications (leakage, bleeding, or stenosis), reoperations, hospital stay	Pooled incidences of anastomotic complications (NS, *p* = 0.821). Leakage: 6.6% vs 4.8% (NS, *p* = 0.518). Hemorrhage: 1.4% vs 1.3% (NS, *p* = 0.431). Stenosis: 2.9% vs 6.8% (NS, *p* = 0.128) Hospital stay: 7 days (NS, *p* = 0.242). Reoperation:7.3% vs 9.6% (NS, *p* = 0.490). Mortality: 0.3% control
Leung et al. (2013) [34]	Colorectal	Left-sided colonic tumor	Laparoscopy without mini-laparotomy; laparoscopy with mini-laparotomy	Stapled (circular; side-to-end, end-to-end)	Air leak test, colonoscopy, histopathologic examination	-	MBP; antibiotic prophylaxis	Operating time, blood loss, length of hospital stay, pain score, wound infection	Operating time: 105 vs. 100 min (*p* = 0.851). Blood loss: 30 vs. 30 ml (*p* = 0.954). Hospital stay: 5 vs. 5 days (*p* = 0.990). Maximum pain score during the first week: 1 vs. 2 (*p* = 0.017). No patients in the HNC group developed wound infection, whereas four patients in the CL group did so (*p* = 0.005)
Khromov et al. (2013) [35]	Colorectal	Elective colorectal resection with the Biodynamix ColonRing^TM^ compression anastomosis ring	Laparotomy; laparoscopy	Compression (Biodynamix ColonRing™)	Air leak test, betadine (povidone-iodine) instillation, macroscopic and histological assessment of the integrity of the doughnuts, proctoscopic examination, CT scan	Additional reinforcing sutures	Enemas; MBP (oral polyethylene glycol); intravenous antibiotic prophylaxis (cephalosporins, garamicin)	Anastomotic leakage, hospital stay, time to first passage of flatus and stool and to oral intake, anastomotic stenosis, wound infection, reoperation	Duration of surgery: 120 min. Anastomotic time: 14.8 min. Height of anastomosis from the anal verge: 18.2 cm. Time to the passage of first flatus and first stool: 2.4 and 3.5 days, respectively. Hospital stay: 7.3 days. Anastomotic leakage: 5%. Wound infection: 5%. No anastomotic stricture. There was one postoperative death (unrelated to an anastomotic complication)
Ruiz-Tovar et al. (2012) [36]	Colon	Right-sided colon cancer and elective surgery	Laparotomy	Stapled (linear, GIA80™; side-to-side/end-to-end); handsewn (2 layers; continuous; 3–0 polyglactyn; side-to-side)	CT scan	-	No bowel preparation; antibiotic prophylaxis (metronidazole, tobramycin)	Wound infection, intra-abdominal infection, anastomotic leak, hemorrhage, operative time, intra-abdominal abscess, mortality, hospital stay	Intra-abdominal abscesses: 5% in each group. Wound infection: 10% stapled, 7% handsewn. Operative time: 98.8 min stapled, 105.2 min handsewn (*p* = 0.013). Positive cultures were obtained in 79% of the cases after stapled anastomosis and 73% after handsewn
Zurbuchen et al. (2013) [37]	Colon	Stenosing ileitis terminalis in Crohn’s disease who underwent an ileocolic resection	Laparotomy; laparoscopy; conversion	Stapled (linear, TA; side-to-side); handsewn (interrupted, Gambe, 4–0 polyglactin); continuous, monofile; end-to-end)	Endoscopy	-	Anti-inflammatory prophylaxis (prednisolone, mesalazine, immunosuppressive)	Recurrence of Crohn’s disease, bleeding, wound infection, anastomotic leakage, first postoperative stool, hospital stay	Crohn’s disease activity index: 200.5 ± 73.66 side-to-side, 219.6 ± 89.03 end-to-end. Duration of surgery: 126.7 ± 42.8 min side-to-side, 137.4 ± 51.9 min end-to-end. Anastomotic leakage: 6.5% end-to-end. Impaired wound healing: 13.9% side-to-side, 6.5% end-to-end. Hospital stay: 9.9 ± 3.93, 10.4 ± 3.26 days
Bertani et al. (2011) [38]	Colorectal	Radical colorectal resection for malignancy with primary anastomosis	Laparotomy; laparoscopy; robot	Stapled (end‐to‐end, termino‐lateral)	Radiography, bacterial examination, surgery	Eight patients were not able to complete MBP	Enemas; MBP (polyethylene glycol, hydro-electrolyte infusion); Intravenous antibiotic prophylaxis (cefoxitin, metronidazole, gentamicin, clindamycin)	Surgical-site infection (anastomotic leakage, wound infection, intra‐abdominal abscess), extra‐abdominal infection and non‐infectious complications, hospital stay, postoperative day of first bowel movement to gas, mortality, assessment of response by patients undergoing mechanical bowel preparation and by surgeons	Surgical site infection: 14.0% MBP, 17.8% no-MBP (*p* = 0.475); Comparable in low anterior resection (*p* = 1.000), and minimally invasive procedure (*p* = 0.241). No perioperative mortality
Buchberg et al. (2011) [18]	Colorectal	Left-sided colectomy and compression anastomosis with the CAR™ 27 device	Laparotomy; laparoscopy	Compression (NiTi CAR™ 27, end-to-end)	Air leak test, proctoscopic examination (flexible sigmoidoscopy), direct palpation from the abdominal side, surgery	Additional purse string suture	MBP; Intravenous antibiotic prophylaxis	Anastomotic leakage, time to return of bowel function, first postoperative toleration of liquids and solids, intraoperative device failure, bleeding, stricture, wound infection, abscess formation, peritonitis, readmission, reoperation, death, length of surgical procedure, ring expulsion time, and awareness	Minor morbidities: 13%, included one small postoperative abscess requiring antibiotics alone and two postoperative anastomotic strictures requiring balloon dilation. Major morbidities: 4%, included a partial anastomotic dehiscence/leakage requiring the surgical dismantling of the anastomosis and diversion

In the handsewn anastomosis, 146 patients of the 3513 presented dehiscences, 87 died in a total of 3482, 288 out of 3025 were reoperated, 2 out of 252 presented stricture, 36 out of 325 developed wound infection, 8 out of 416 presented intra-abdominal abscess, and 49 showed bleeding in a total of 2815 patients. The mean duration of surgery was 139.92 (112.15–167.70) min in a total of 2937 patients. The mean hospital stay was 13.20 (7.00–19.39) days in 3189 patients.

Regarding the stapled anastomosis, 164 out of the 3079 presented dehiscences, 79 in 2874 patients died, 179 out of 1960 were reoperated, 28 out of 1783 showed bleeding, 11 out of 185 presented stricture, 37 out of 476 developed wound infection, and 10 out of 471 present intra-abdominal abscess. The mean duration of surgery was 141.25 (105.55–176.94) min in a total of 1897 patients. The mean hospital stay was 10.52 (8.58–12.46) days in 2242 patients.

Regarding compression anastomosis, 17 out of the 329 presented dehiscences, 3 patients died in a total of 329, 12 out of the 329 were reoperated, 8 out of the 329 presented bleeding, 3 out of 112 presented stricture, 3 out of 63 developed wound infection, and 8 in the 329 presented intra-abdominal abscess. The mean duration of surgery was 183.47 (163.90–203.04) min for a total of 63 patients. The mean of hospital stay was 11.92 (9.30–14.54) days in 323 patients.

### Meta-analysis of clinical outcomes

The overall pooled results of the primary and secondary outcomes compared in this meta-analysis are summarized in Table 3.

**Table 3:** Statistical analysis of the outcomes

**Outcome**	**Number of studies**	**Number of patients**	**Events per 100 patients or MRAW [95% CI]**	***p***	***I***^**2**^**(%)**	**Chi-square test (X**^**2**^**),** ***p***
**Primary outcomes**						
Dehiscence	16	6921	4.69 [3.56; 5.82]	0.81	63	< 0.01
Mortality	12	6685	1.47 [0.75; 2.19]	0.78	77	< 0.01
Reoperation	10	5255	6.84 [4.74; 8.95]	< 0.01	86	< 0.01
Bleeding	10	4986	1.53 [0.57; 2.49]	0.73	0	0.65
Stricture	6	549	2.52 [0.00; 5.25]	0.17	44	0.11
**Secondary outcomes**						
Wound infection	9	864	7.26 [4.71; 9.81]	0.31	35	0.12
Intra-abdominal abscess	9	1216	1.74 [0.37; 3.11]	0.91	4	0.40
Duration of surgery (minutes)	10	4897	146.80 [124.05; 169.54]	0.02	99	< 0.01
Length of hospital stay (days)	14	6532	11.49 [9.42; 13.56]	0.56	99	< 0.01

#### Primary outcomes

**Fig. 2:**
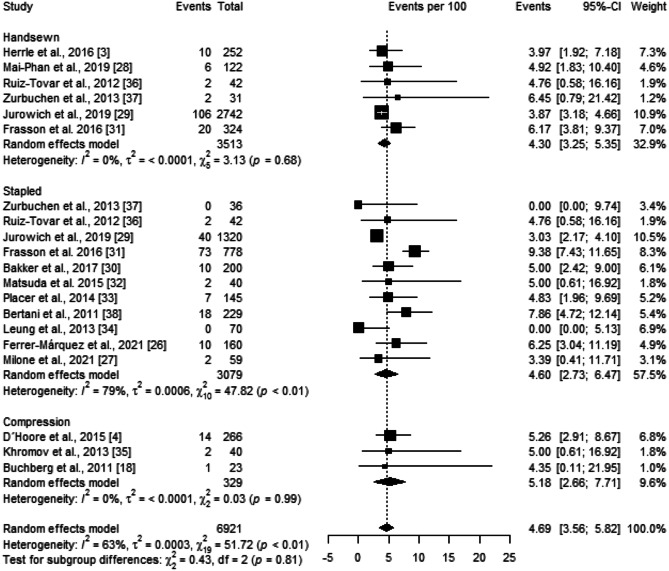
Forest plot of the proportion of dehiscence grouped by anastomotic technique (handsewn, stapled, or compression). Values are presented as proportions with a 95% CI

The dehiscence rate across all studies included in the quantitative analysis was 4.69 [3.56; 5.82]%. Subgroup analysis showed no significant differences between anastomotic techniques (*p* = 0.81) (Fig. 2). However, there is substantial heterogeneity among the studies within the stapled group (${\chi }_{10}^{2}=47.82,$ *p* < 0.01, *I* = 79%). Sensitivity analysis was performed by removing each study included in the meta-analysis individually. This exclusion did not substantially affect heterogeneity and had little effect on the statistical analysis of dehiscence rate. Moreover, Egger’s test demonstrated no publication bias (*p* = 0.28).

**Fig. 3:**
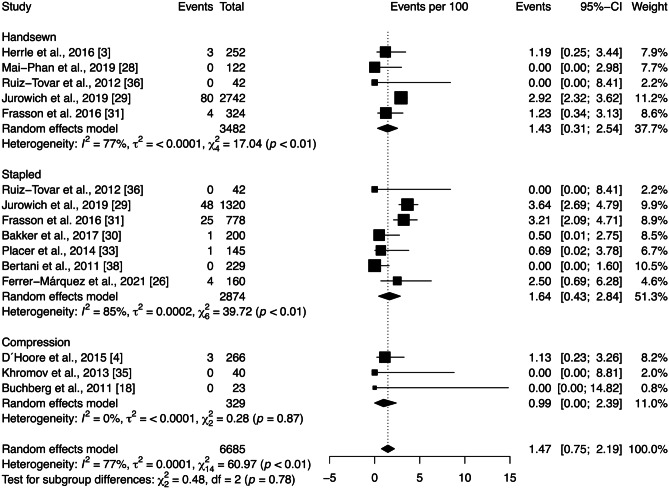
Forest plot of deaths per 100 patients grouped by anastomotic technique (handsewn, stapled, or compression). Values are presented as proportions with a 95% CI

The mortality rate across all studies included in the quantitative analysis was 1.47 [0.75; 2.19]%. Subgroup analysis showed no significant differences between anastomotic technique groups (*p* = 0.78) (Fig. 3). However, there is substantial heterogeneity among the studies within the handsewn and stapled anastomotic techniques (${\chi }_{4}^{2}=17.04,$ *p* < 0.01, *I* = 77%; ${\chi }_{6}^{2}=39.72,$ *p* < 0.01, *I* = 85%, respectively). Sensitivity analysis was performed by removing each study included in the meta-analysis individually. The heterogeneity was substantially decreased in the handsewn group after the exclusion of the Jurowich et al. [29] study. However, this change had little effect on the statistical analysis of the mortality rate. Moreover, Egger’s test demonstrated no publication bias (*p* = 0.21).

**Fig. 4:**
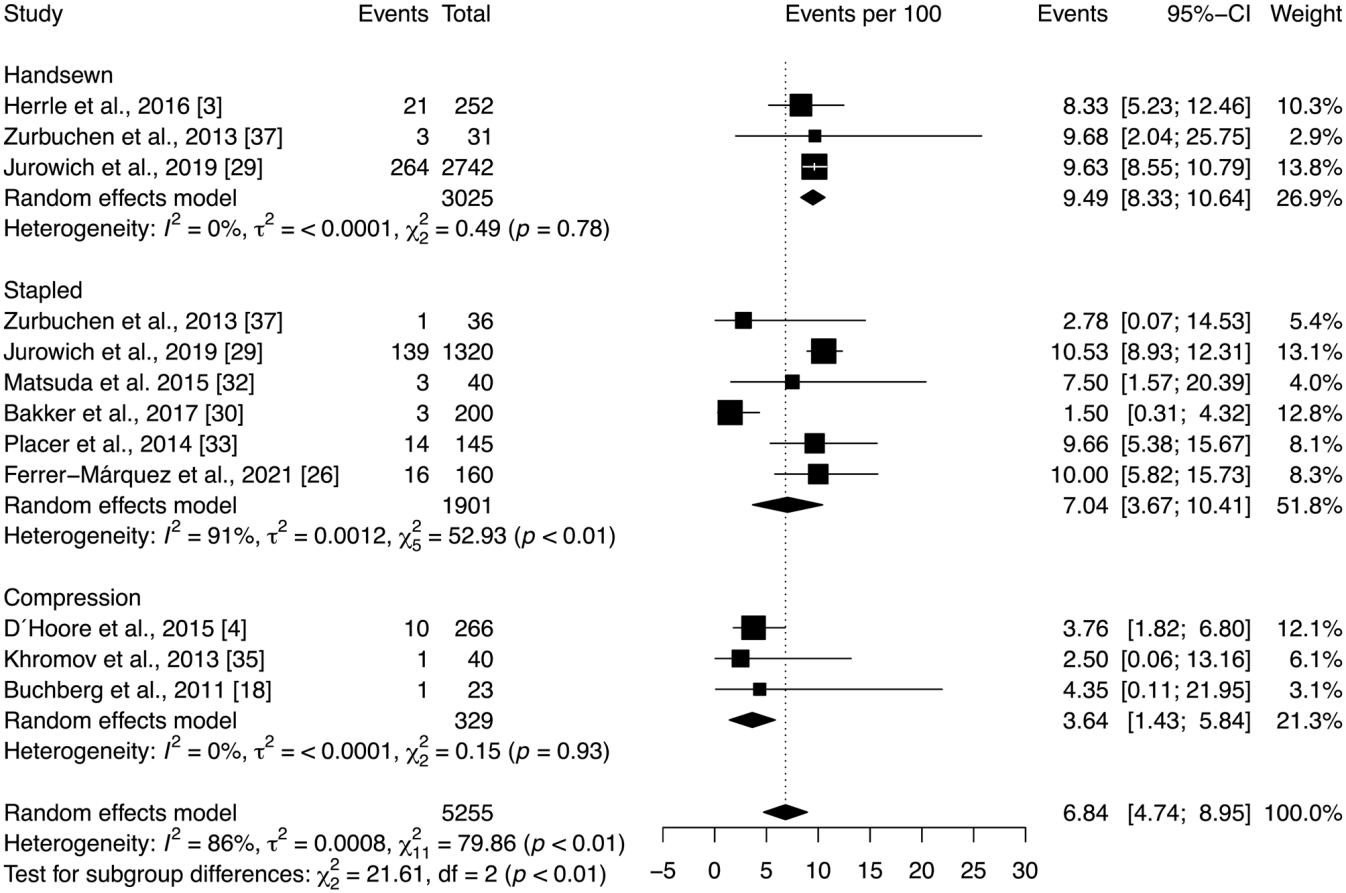
Forest plot of the proportion of reoperation grouped by anastomotic technique (handsewn, stapled, or compression). Values are presented as proportions with a 95% CI

The reoperation rate across all studies included in the quantitative analysis was 6.84 [4.74; 8.95]%. According to Fig. 4, subgroup analysis showed significant differences between anastomotic techniques (*p* < 0.01), with the compression technique reporting the lowest reoperation rate (3.64 [1.43; 5.84]%) and the handsewn the higher (9.49 [8.33; 10.64]%). However, there is substantial heterogeneity among the studies within the stapled group (${\chi }_{5}^{2}=52.93,$ *p* < 0.01, *I* = 91%). Sensitivity analysis was performed by removing each study included in the meta-analysis individually. The exclusion of each study did not substantially affect heterogeneity and had little effect on the statistical analysis of the reoperation rate. Moreover, Egger’s test demonstrated no publication bias (*p* = 0.42).

**Fig. 5:**
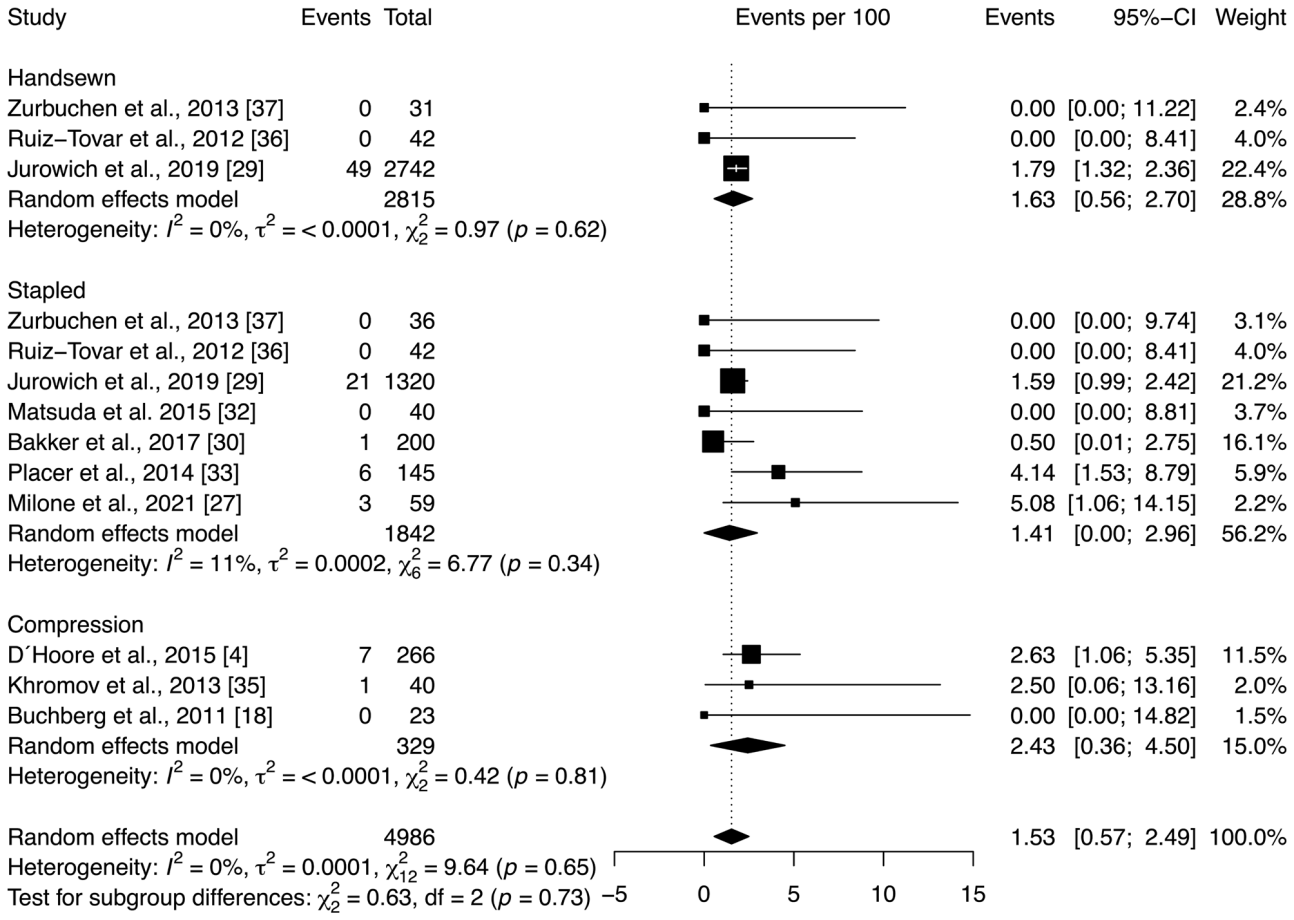
Forest plot of the proportion of bleeding grouped by anastomotic technique (handsewn, stapled, or compression). Values are presented as proportions with a 95% CI

The bleeding rate across all studies included in the quantitative analysis was 1.53 [0.57; 2.49]%. Subgroup analysis showed no significant differences between anastomotic techniques (*p* = 0.73) (Fig. 5). No significant heterogeneity among the studies was observed (${\chi }_{12}^{2}=9.64,$ *p* = 0.65, *I* = 0%). Moreover, Egger’s test demonstrated no publication bias (*p* = 0.61).

**Fig. 6:**
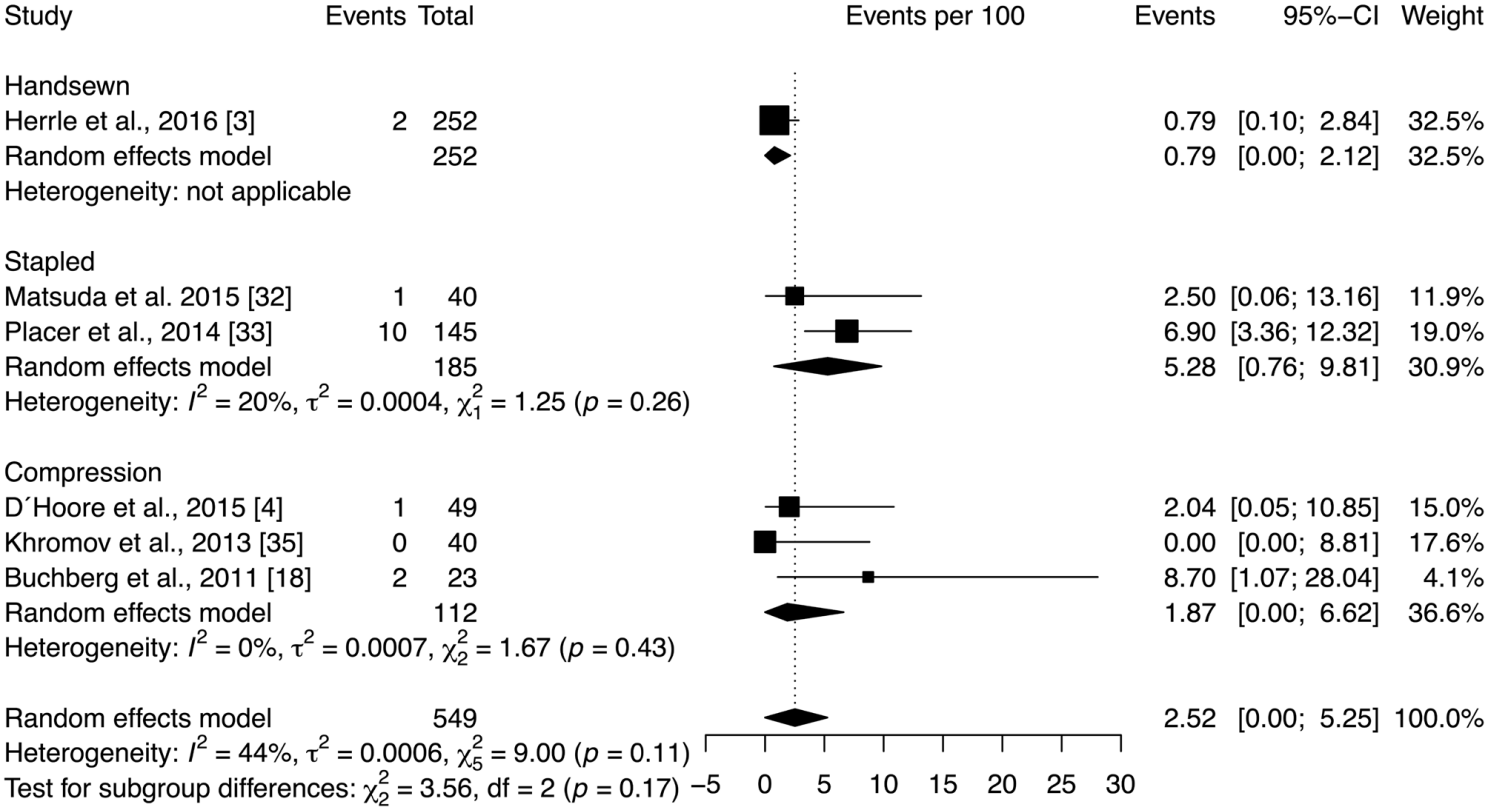
Forest plot of the proportion of stricture grouped by anastomotic technique (handsewn, stapled, or compression). Values are presented as proportions with a 95% CI

The stricture rate across all studies included in the quantitative analysis was 2.52 [0.00; 5.25]%. Moderate heterogeneity among the studies (${\chi }_{5}^{2}=9.00,$ *p* < 0.11, *I* = 44%) was observed. Subgroup analysis showed no significant differences between anastomotic techniques (*p* = 0.17) (Fig. 6). Sensitivity analysis was performed by removing each study included in the meta-analysis individually. The heterogeneity was substantially decreased after the exclusion of the Placer et al. [33] study. However, this change had little effect on the statistical analysis of proportion of stricture.

#### Secondary outcomes

**Fig. 7:**
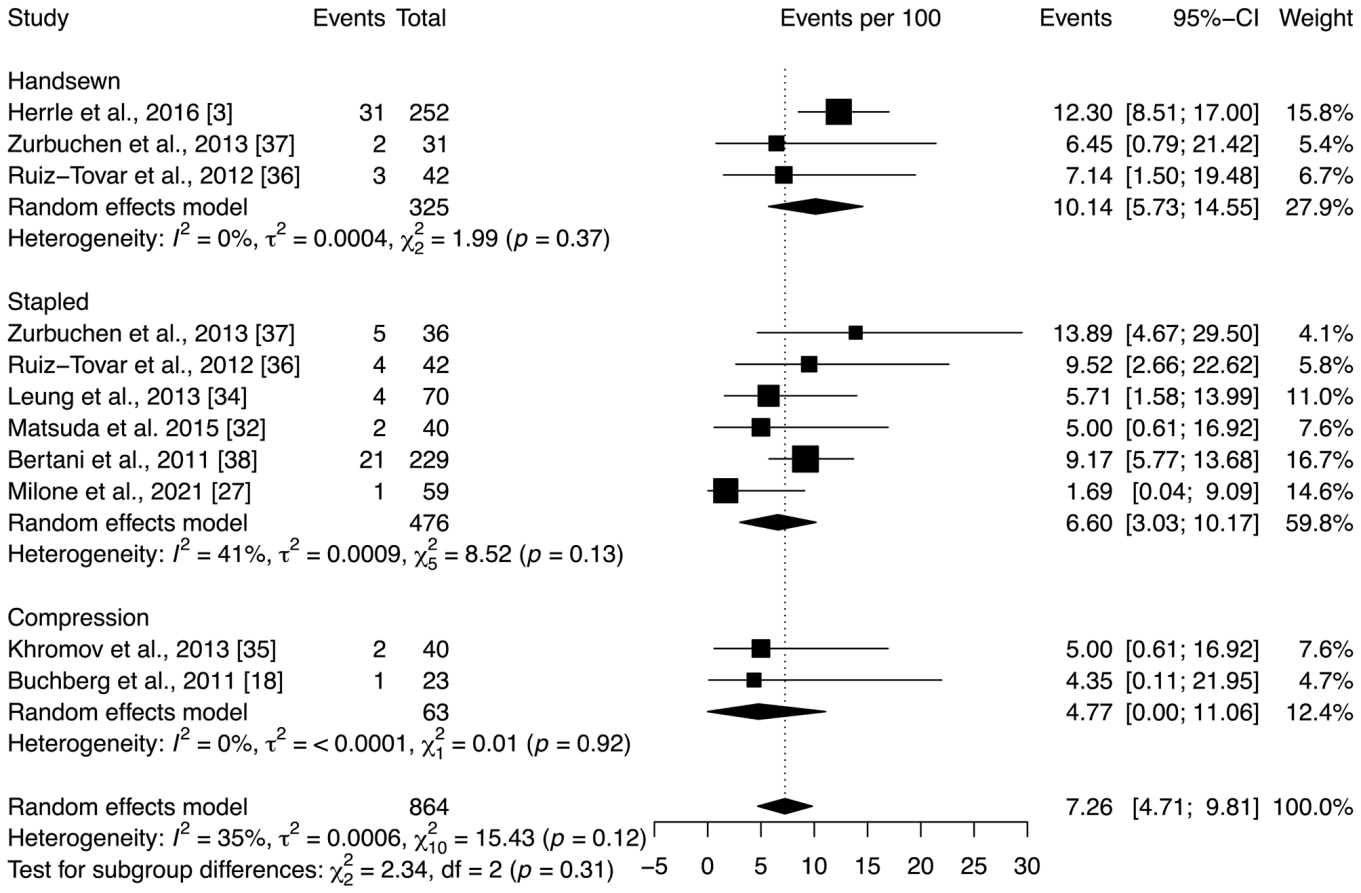
Forest plot of the proportion of wound infection grouped by anastomotic technique (handsewn, stapled, or compression). Values are presented as proportions with a 95% CI

The wound infection rate across all studies included in the quantitative analysis was 7.26 [4.71; 9.81]%. Subgroup analysis showed no significant differences between anastomotic techniques (*p* = 0.31) (Fig. 7). Moderate heterogeneity among the studies was observed (${\chi }_{10}^{2}=15.43,$ *p* = 0.12, *I* = 35%). Sensitivity analysis was performed by removing each study included in the meta-analysis individually. The heterogeneity was substantially decreased after the exclusion of the Herrle et al. [3] and Milone et al*.* [27] studies. However, this change had little effect on the statistical analysis of wound infection rate. Moreover, Egger’s test demonstrated no publication bias (*p* = 0.68).

**Fig. 8:**
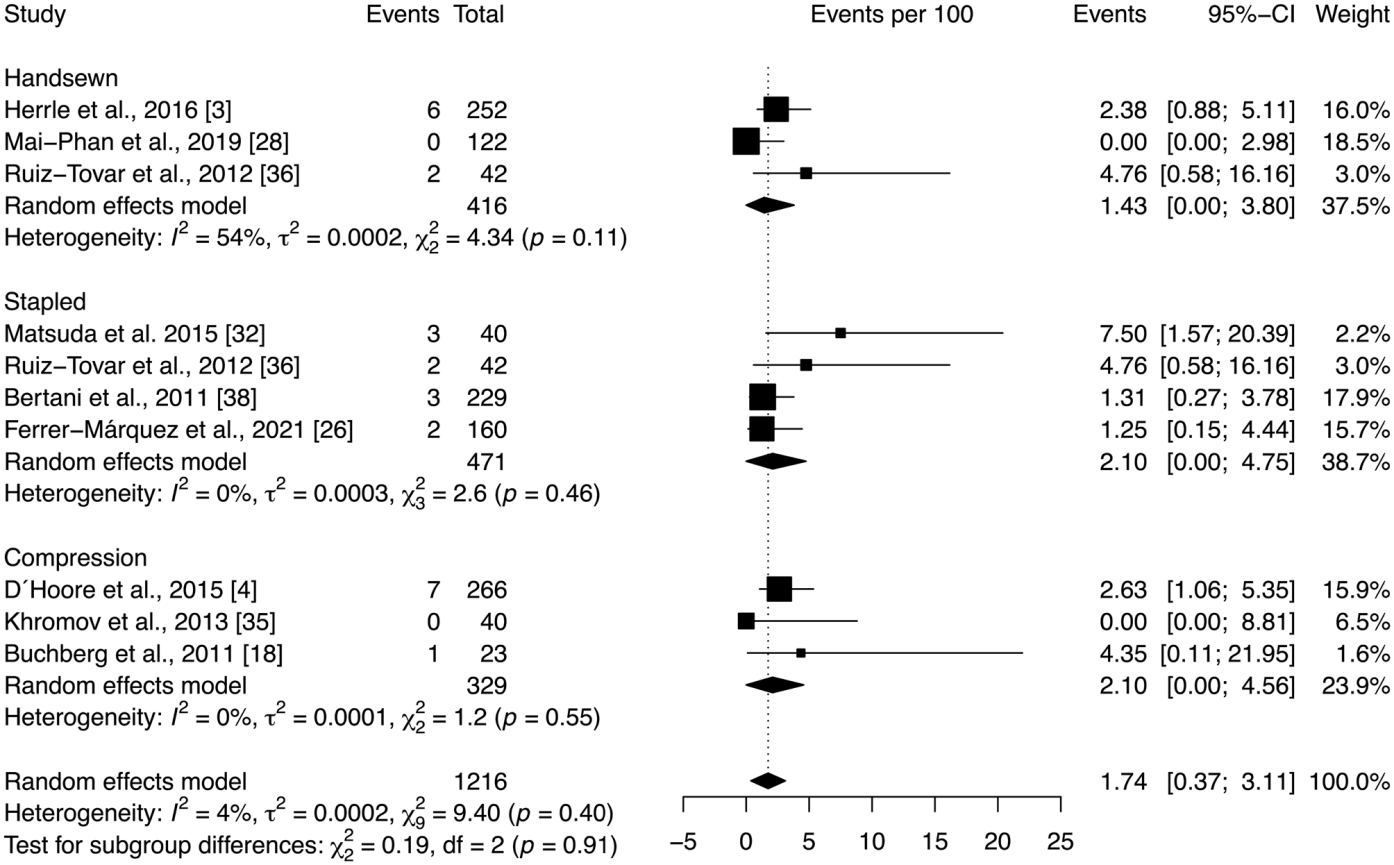
Forest plot of the proportion of intra-abdominal abscess grouped by anastomotic technique (handsewn, stapled, or compression). Values are presented as proportions with a 95% CI

The intra-abdominal abscess rate across all studies included in the quantitative analysis was 1.74 [0.37; 3.11]%. Subgroup analysis showed no significant differences between anastomotic techniques (*p* = 0.91) (Fig. 8). Low heterogeneity among the studies was observed (${\chi }_{9}^{2}=9.40,$ *p* = 0.40, *I* = 4%).

**Fig. 9:**
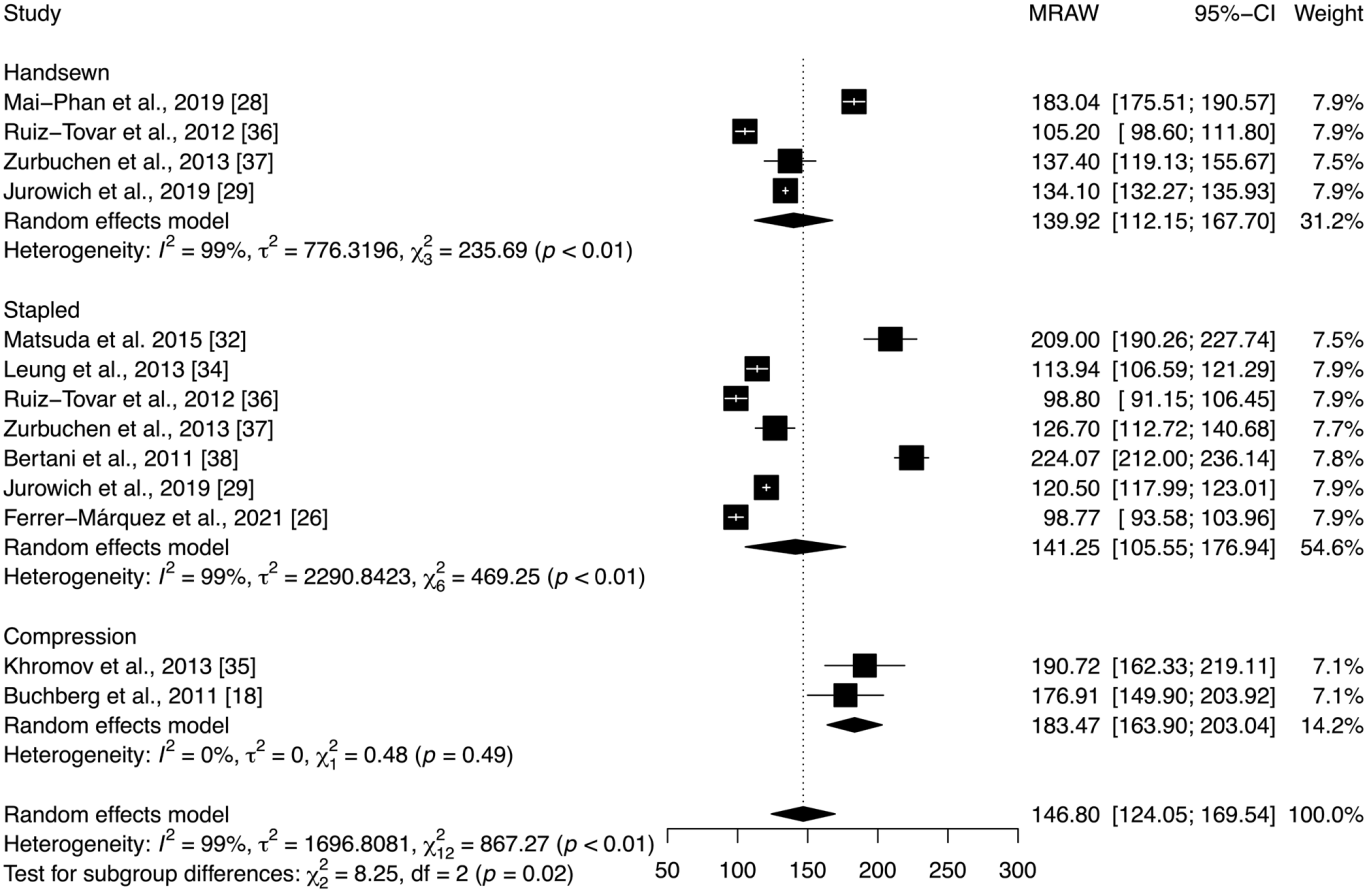
Forest plot of the mean duration of surgery grouped by anastomotic technique (handsewn, stapled, or compression). Values are presented as means with a 95% CI

The mean duration of surgery across all studies included in the quantitative analysis was 146.80 [124.05; 169.54] min. According to Fig. 9, subgroup analysis showed significant differences between anastomotic techniques (*p* = 0.02), with the compression technique reporting the longer time to perform the surgery (183.47 [163.90–203.04] min). However, there is substantial heterogeneity among the studies within the stapled and handsewn groups (${\chi }_{6}^{2}=469.27,$ *p* < 0.01, *I* = 99%; ${\chi }_{3}^{2}=235.68,$ *p* < 0.01, I = 99%, respectively). Sensitivity analysis was performed by removing each study included in the meta-analysis individually. The exclusion of each study did not substantially affect heterogeneity and had little effect on the statistical analysis of the duration of surgery. Moreover, Egger’s test demonstrated no publication bias (*p* = 0.44).

**Fig. 10:**
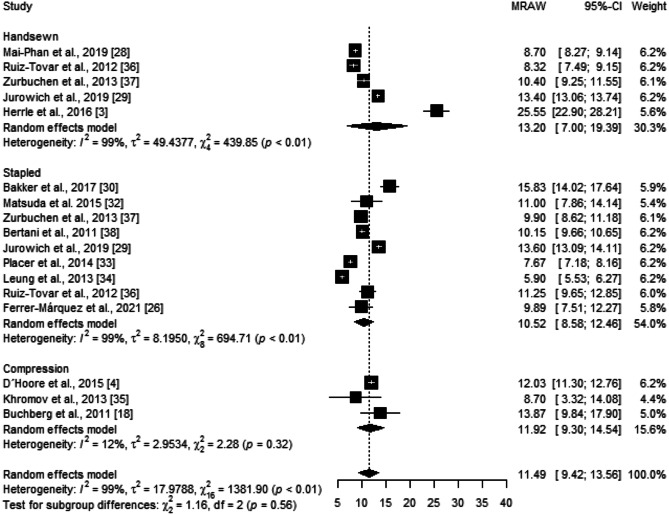
Forest plot of the mean of hospital stay grouped by anastomotic technique (handsewn, stapled, or compression). Values are presented as means with a 95% CI

The mean of hospital stay across all studies included in the quantitative analysis was 11.49 [9.42; 13.56] days. Subgroup analysis showed no significant differences between anastomotic techniques (*p* = 0.56) (Fig. 10). However, there is substantial heterogeneity among the studies within the stapled and handsewn groups (${\chi }_{8}^{2}=694.71,$ *p* < 0.01, *I* = 99%; ${\chi }_{4}^{2}=439.85,$ *p* < 0.01, *I* = 99%, respectively). Sensitivity analysis was performed by removing each study included in the meta-analysis individually. The exclusion of each study did not substantially affect heterogeneity and had little effect on the statistical analysis of hospital stay. Moreover, Egger’s test demonstrated no publication bias (*p* = 0.46).

## Discussion

This systematic review and meta-analysis compares three different anastomotic techniques, namely handsewn, stapled, and compression, independently. Our analysis demonstrated that, when a colonic and rectal surgery is performed, among the wide variety of complications that may occur, the wound infection showed a high prevalence (7.26% [4.71; 9.81]%), followed by the need of reoperation and dehiscence. It was also demonstrated no significant differences among handsewn, stapled, and compression techniques regarding the occurrence of dehiscence, deaths, bleeding, stricture, wound infection, intra-abdominal abscess, and in the length of hospital stay. However, differences were found regarding the reoperation rates, with the handsewn technique reporting the highest rate and the compression the lowest. Statistically significant differences were also found in the time needed for surgery. Regarding this outcome, the compression technique required more time, being the handsewn the fastest technique.

Although the procedures of each technique differ, they are quite similar concerning postoperative outcomes, and doubts still exist regarding which procedure should be adopted. In general, the literature also found no differences between the techniques for most of the outcomes analyzed. Lustosa et al. [39] did not found differences between the handsewn and stapled techniques regarding mortality, dehiscence, hemorrhage, reoperation, wound infection, and hospital stay. However, differences were found in stricture with stapled technique reporting a higher incidence, and the handsewn technique requiring more anastomotic time. MacRae et al*.* [20] also found that stricture was more common in stapled technique than in handsewn technique. Despite that, no differences were found in mortality and clinical and radiologic leakage rates between the two techniques. Likewise, Neutzling et al. [2] found no significant differences between the handsewn and stapled techniques regarding dehiscence rates, but also observed significant stenosis in patients who underwent stapled anastomosis comparing with handsewn. In turn, Slesser et al. [40] compared compression with handsewn/stapled anastomosis and did not find significant differences regarding mortality, anastomotic leakage, stricture, length of surgery, and wound-related, but a shorter postoperative stay was associated with compression technique. Some authors [35, 41] reported that in compression anastomosis, there is no retention of a foreign body at the anastomotic site and, therefore, a reduced probability of late luminal narrowing. Other studies [9, 42] reported that compression anastomosis is associated with lower stricture rates when compared to other techniques. For this reason, a lower incidence of wound infections should be expected [35]. For others [20], the intraoperative technical problems were more likely to occur with stapled than handsewn anastomosis, but our results do not corroborate this observation. Khromov et al*.* [35] demonstrated that compression technique presents anastomotic complications comparable to the stapled. Kracht et al. [43] suggested that in stapled anastomosis, there is less intra-operative septic contamination since only small holes are opened to introduce the stapler; whereas, in the handsewn anastomosis, the entire lumen of the colon is exposed. Ruiz-Tovar et al. [36] consider that the size of the opening of the lumen does not have a determining role in peritoneal contamination. This may be greater in the stapled anastomosis as a result of the fecal material that is released when removing the stapler from the colon’s lumen, but this finding was not observed in our results.

Taken together, our findings demonstrated that the compression technique takes longer, but it can bring a better prognosis in terms of future complications that lead to the need of reoperation. However, possible study limitations may affect the quality of the results obtained. The first limitation is related to the lack of direct comparison studies between handsewn, stapled, and compression anastomosis. Thus, this analysis included singular studies of each of the anastomotic techniques, so that some types of variability in the definition of outcomes, period of follow-up, and surgical procedures may occur. Compression anastomosis was limited to the use of a single device, and few studies still exist using this surgical procedure. In addition, there is still a set of variables associated with clinical practice, ranging from different clinical conditions of the patients, surgeons’ skills, type of surgery performed, as well as anastomotic configurations. The duration of the surgery is influenced by all surgical steps performed and that may differ between studies and patients. Thus, ideally, only the time required to perform the anastomosis would be considered, but these data are scarce in the articles. For the analysis of wound infection, data mentioning surgical site infection were also excluded due to the impossibility to know accurately whether it was a superficial infection restricted to the wound site or not. The results obtained are still influenced by the method used to diagnose complications, combined with the personal interpretation that some cases may have. Furthermore, certain characteristics of the studies can also have their contribution, such as the existence or not of randomization and the type of existing blind. All these study limitations should be considered when adopting our findings to the clinical practice.

## Conclusions

The results obtained from this systematic review and meta-analysis did not find sufficient evidences to conclude which of the anastomotic techniques is more efficient and promotes fewer postoperative complications. The differences found were limited to the rate of reoperations, with compression anastomosis reporting the lowest rate and the handsewn anastomosis the highest. Despite this, surgeries with compression anastomosis required more time, with the handsewn being the fastest technique. Therefore, it is not possible to infer conclusions about which procedure is more appropriate for anastomosis.

## Data Availability

The data analyzed in this study will be made available upon reasonable request.
